# Experimental, Computational, and Dimensional Analysis of the Mechanical Performance of Fused Filament Fabrication Parts

**DOI:** 10.3390/polym13111766

**Published:** 2021-05-27

**Authors:** Iván Rivet, Narges Dialami, Miguel Cervera, Michele Chiumenti, Guillermo Reyes, Marco A. Pérez

**Affiliations:** 1 International Center for Numerical Methods in Engineering (CIMNE), Campus Norte UPC, Technical University of Catalonia, 08034 Barcelona, Spain; narges.dialami@upc.edu (N.D.); miguel.cervera@upc.edu (M.C.); michele.chiumenti@upc.edu (M.C.); 2 IQS School of Engineering, University Ramon Llull, Via Augusta 390, 08017 Barcelona, Spain; guillermo.reyes@iqs.url.edu (G.R.); marcoantonio.perez@iqs.edu (M.A.P.)

**Keywords:** additive manufacturing, material characterization, transverse isotropy, adhesion, mechanical properties, computational homogenization

## Abstract

Process parameters in Additive Manufacturing (AM) are key factors in the mechanical performance of 3D-printed parts. In order to study their effect, a three-zone model based on the printing pattern was developed. This modelization distinguished three different zones of the 3D-printed part, namely cover, contour, and inner; each zone was treated as a different material. The cover and contour zones were characterized via uniaxial tensile tests and the inner zones via computational homogenization. The model was then validated by means of bending tests and their corresponding computational simulations. To reduce the number of required characterization experiments, a relationship between the raw and 3D-printed material was established by dimensional analysis. This allowed describing the mechanical properties of the printed part with a reduced set of the most influential non-dimensional relationships. The influence on the performance of the parts of inter-layer adhesion was also addressed in this work via the characterization of samples made of Polycarbonate Acrylonitrile Butadiene Styrene (ABS/PC), a polymeric material well known for its poor adhesion strength. It was concluded that by using this approach, the number of required testing configurations could be reduced by two thirds, which implies considerable cost savings.

## 1. Introduction

In recent years, the field of the Additive Manufacturing (AM) or 3D printing has experienced an uninterrupted rise [1]. This technology consists of adding material in a layer-by-layer fashion to fabricate the final three-dimensional part, normally from a CAD model. The main causes of its growth are its design freedom, which enables the production of complex-shaped components that are hardly obtainable by other manufacturing methodologies, as well as its cost-effectiveness [2]. The AM technologies can be classified based on many criteria [3]. According to the deposition method, a wide variety of printing technologies exist: Multi-Jet Fusion (MJF) [4], Selective Laser Melting (SLM) [5], Electron Beam Melting (EBM) [6], and Fused Filament Fabrication (FFF) [7], among others. This work was focused on FFF, also called Fused Deposition Modeling (FDM), which is one of the best established AM technologies.

In FFF, a thermoplastic filament is heated a few degrees above its glass transition temperature and extruded through the heated nozzle, then placed to form a layer. This newly deposited layer solidifies and bonds with previously deposited ones, forming the desired 3D geometry [8]. The main setting parameters are sample orientation, printing pattern, and layer thickness. The most remarkable features of FFF encompass reduced weight and material use to operating with a wide range of polymeric materials [9]. Furthermore, FFF parts are extensively used for rapid prototyping in many industries such as aerospace, automobile, medical, electronics, etc. [10].

In contrast, one of the key characteristics of AM materials, which is not an exception for FFF, is that the properties of the final parts differ from those of their raw material. It has been shown that these properties strongly depend on the manufacturing process parameters [11,12], but this dependency is not yet fully understood. This lack of knowledge regarding the process properties’ dependency increases the printing randomness and causes a dispersion of the mechanical properties of the final printed parts [13], making the FFF technology less attractive from the industrial point of view.

This issue has been extensively studied in recent years. Related investigations can be basically classified between those that study the dependency and the influence of the process parameters on the final properties of the printed part [14,15,16,17,18,19,20,21,22] and those that explicitly characterize these properties for a set of specific process parameters [23,24,25]. Their main findings conclude that, on the one hand, the stiffness in the build direction is the weakest one due to inter-layer unions (also known as the inter-layer adhesion issue) and, on the other hand, the resistance in the direction of the extruded filament is higher than the one of the intra-layer unions. Moreover, the properties of FDM parts in the direction perpendicular to the deposition orientation have been found to be more dependent on the bonding effect than on the properties of the raw material itself [26]. Finally, several rheology-centric investigations have been carried out [27,28,29,30]. These investigations express the need for careful rheological knowledge to achieve stronger 3D-printed parts, highlight the importance of viscosity, temperature, pressure, and shear rate in material extrusion processes [31]. A common aspect in all these studies is the fact that the whole 3D-printed part is considered as a single material that shows an anisotropic behavior. Since multiple printing patterns are employed during the manufacturing process, this approximation does not truly represent the underlying physics of the case.

**Figure 1:**
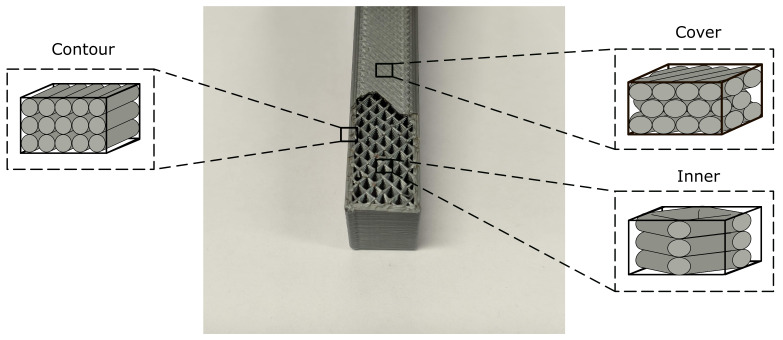
Zone distinction of a 3D-printed part.

In this work, the mechanical behavior of FFF parts was represented by a three-zone model distinguishing among the following zones: cover, contour, and inner (Figure 1). Each zone corresponded to a different printing pattern: covers were printed with crossed filaments; contours were printed with aligned filaments; and inner zones could adopt several different patterns, the most common being the ±45° pattern. The distinction between covers (horizontal surfaces) and contours was made based on the surface angle with respect to the horizontal printing plane: covers had a slope below 30° and contours above that threshold. Two different types of inner zones existed: in-fill and lattice structures. The in-fill type refers to a standard pattern defined by both its geometry and air gap, while the lattice consists of a structure made of a periodically repeated unit cell.

In previous papers [32,33], this three-zone model was used in order to characterize the properties of printed parts for Polylactic Acid (PLA) and General Purpose Acrylonitrile Butadiene Styrene (ABS/GP), respectively. The novelty of this paper was to extend this background to Polycarbonate Acrylonitrile Butadiene Styrene (ABS/PC) and to use dimensional analysis to reduce the number of required experiments and to generalize the non-dimensional mechanical properties for different materials. The major advantages of using dimensional analysis are the reduction of the number of parameters involved in the problem, the possibility to extrapolate the results to non-tested materials, and a better understanding of the dependencies among the involved parameters [34]. The study of the inter-layer adhesion effect is of special interest, which depends to a large extent on the material through temperature-dependent properties [35,36]. Thus, this work also aimed at a better understanding of the adhesion phenomena and the identification of which mechanical properties were adhesion independent. ABS/PC was the chosen raw polymer used in this paper because polycarbonate materials are known to exhibit poor adhesion strength [37].

The paper is organized as follows. First, Section 2 presents the three-zone constitutive model. Section 3 describes the experimental procedures followed (i) to characterize, through tensile tests, the contour and top and bottom covers and (ii) to validate, through bending tests, the obtained data. In Section 4, the solution strategy used for the computational simulations employed to obtain the inner zone properties is explained. Section 5 discusses the obtained results, and Section 6 introduces the proposed dimensional analysis of the three-zone model. Finally, the paper’s concluding remarks and possible future research lines are presented in Section 7.

## 2. Material Characterization

The raw material used in this paper was ABS/PC for 3D printing manufactured by ELIX Polymers, which behaves as an isotropic material with mechanical properties $E_{raw}=2340$ MPa and $v_{raw}=0.36$ [38,39]. Polymers’ raw properties strongly depend on factors such as the manufacturing process, polymer chain type, or additives and, therefore, can vary among different providers [40]. Nevertheless, due to the AM procedure, final 3D-printed parts do not behave isotropically; they exhibit noticeable orthotropic mechanical properties [41].

**Figure 2:**
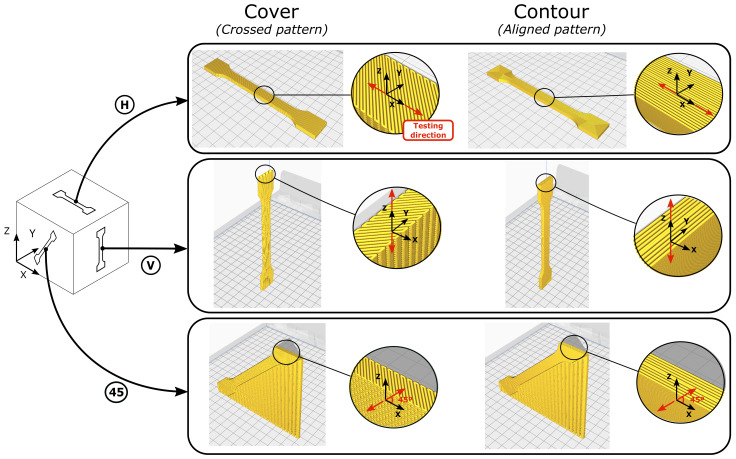
Orientation of the dog bone specimens.

For the characterization of printed parts, in this paper, the component was divided into three different zones to treat them as *different materials*. It is convenient to define the standard coordinate system for a 3D-printing process (shown in Figure 2) where XY denotes the horizontal printing plane and Z is the vertical direction (also called the printing direction).

To fully characterize each zone, either the constitutive tensor C or its inverse, the compliance tensor D, must be obtained. An orthotropic elastic material requires nine material properties for its complete characterization. However, if the previous distinction among zones is considered, the material properties of each separate region can be considered transversely isotropic, meaning that the material presents an isotropic behavior in a certain plane. Due to the symmetry presented in this plane, the required number of material parameters is reduced from nine to five for each zone. For an XY isotropic material, the transversely isotropic compliance tensor, using Voigt’s notation, is:

(1) $$D=\left[\begin{array}{cccccc} \frac{1}{E} & -\frac{\nu_{xy}}{E} & -\frac{\nu_{zx}}{E_{z}} & 0 & 0 & 0\\ -\frac{\nu_{xy}}{E} & \frac{1}{E} & -\frac{\nu_{zx}}{E_{z}} & 0 & 0 & 0\\ -\frac{\nu_{zx}}{E_{z}} & -\frac{\nu_{zx}}{E_{z}} & \frac{1}{E_{z}} & 0 & 0 & 0\\ 0 & 0 & 0 & \frac{2(1+\nu_{xy})}{E} & 0 & 0\\ 0 & 0 & 0 & 0 & \frac{1}{G} & 0\\ 0 & 0 & 0 & 0 & 0 & \frac{1}{G} \end{array}\right]$$

where $E_{i}$ accounts for Young’s modulus in the *i* direction, $\nu_{ij}$ for Poisson’s ratio that describes the relation between the deformations in the *j* and *i* directions when a load is applied in the *i* direction, and $G_{ij}$ for the shear modulus in direction *j* in the plane normal to direction *i*. Note that for the XY transversely isotropic tensors are $E=E_{x}=E_{y}$, $\nu_{zy}=\nu_{zx}$, $G_{xy}=E/\left(2\left(1+\nu_{xy}\right)\right)$, and $G=G_{xz}=G_{yz}$.

The isotropic plane for the cover zone is the printing plane (XY), while for the contour zone, it is the one perpendicular to the filament (which depends on the shape of the part). Because of this, it is convenient to adapt the nomenclature shown in Equation(1) for each respective zone as shown in Table 1. For the cover case, $E_{⊥}$ and $E_{iso}$ are Young’s modulus in the direction perpendicular to the isotropic plane (printing plane) and in the isotropic plane, respectively, ν and $\nu_{iso}$ Poisson’s ratio in the printing direction and in the plane of isotropy, and *G* and $G_{iso}$ the shear modulus in the printing direction and in the plane of isotropy. For the contour zone, $E_{\|}$ relates to Young’s modulus in the direction parallel to the filament (X direction in the case shown in Table 1), and the remaining parameters present an analogous description.

The inner zone is also considered a transversely isotropic material, XY being its isotropic plane. Thus, its compliance tensor matches the one in Equation (1).

## 3. Experimental Procedure

In this section, the experiments performed to fully characterize the ABS/PC material are explained in detail. Uniaxial tests were performed in order to obtain the mechanical properties of both the cover and contour zones, while bending tests were used to validate the obtained properties. All the specimens were fabricated using a BCN3D Sigmax printer with the printing parameters shown in Table 2.

### 3.1. Uniaxial Tensile Tests

**Figure 3:**
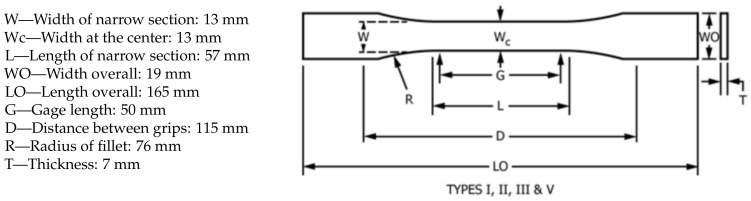
Dog bone specimen dimensions.

Uniaxial tests were performed as per ASTM D638 [42], and multiple dog bone parts were printed according to the standard Specimen Type I shown in Figure 3. All the samples for the tensile tests were printed without contours and covers.

Six different configurations were printed, varying their printing orientation and printing pattern. To ensure repeatability, three samples for each case were produced, with a total of 18 printed samples. For the characterization of the cover, three different dog bones were manufactured using the cross-pattern: 1 in the horizontal plane (H-Cover), 1 aligned with the Z direction (V-Cover), and 1 at 45°(45-Cover). Regarding the contour, three additional dog bones were produced using the aligned pattern: 1 in the horizontal plane (H-Contour), 1 aligned with the Z direction (V-Contour), and 1 with a 45° slope with respect to the XY plane (45-Contour). The purpose of the 45° configurations was the calculation of the shear modulus following the procedure detailed below in this section. The printing patterns for the different orientations of the dog bone specimens are shown in detail in Figure 2.

An initial strain rate of 0.1 mm/(mm·min) was selected, and a testing speed of 5 ± 1.25 mm/min was set for the tensile test according to Specimen Type I for rigid and semi-rigid samples [42]. The yield strength was identified by means of the offset method for determining yield strength considering an offset value of 0.1% [40].

From these tests, the Young and shear modulus of both the cover and contour zones were obtained. Poisson’s ratios were considered to be the same as from the raw material ($\nu_{raw}=0.36$), since it was shown in previous works [32,33] that this assumption has a minor influence on the final results.

The sequence for obtaining the cover zone properties defined in Section 2 was:

- Young’s modulus in the printing direction $E_{⊥}$ was obtained from the V-Cover specimen;
- Young’s modulus in the plane of isotropy $E_{iso}$ was obtained from the H-Cover specimen;
- The shear modulus in the printing direction *G* was obtained from the 45-Cover specimen with:
  (2) $$G=\frac{E_{1}}{2(1+\nu_{12})}$$
  where 1 is the applied load direction and 2 any direction on its perpendicular plane;
- The shear modulus in the plane of isotropy $G_{iso}$ was obtained with:
  (3) $$G_{iso}=\frac{E_{iso}}{2(1+\nu_{iso})}$$

For the contour zone:

- Young’s modulus in the direction parallel to the filament $E_{\|}$ was obtained from the H-Contour specimen;
- Young’s modulus in the plane of isotropy $E_{iso}$ was obtained from the V-Contour specimen;
- The shear modulus in the direction parallel to the filament *G* was obtained from the 45-Contour specimen with Equation (2);
- The shear modulus in the plane of isotropy $G_{iso}$ was obtained from Equation (3).

### 3.2. Bending Tests

**Figure 4:**
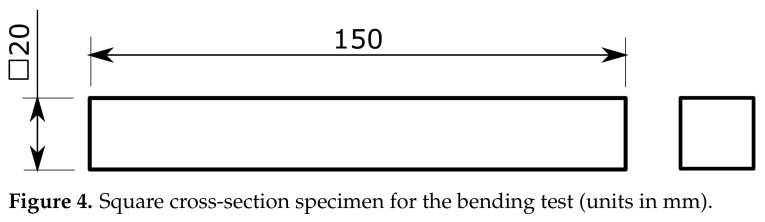
Square cross-section specimen for the bending test (units in mm).

Following ASTM D790 [43], several square cross-section parts (Figure 4) were manufactured and tested under three-point bending conditions with a span between supports equal to 150 mm. The specimens were 150 mm long with a square cross-section of 20 × 20 mm^2^. All samples for the bending tests were printed with contours and covers with a thickness equal to 1 mm, and the in-fill structure was conformed by a ±45° linear pattern.

**Figure 5:**
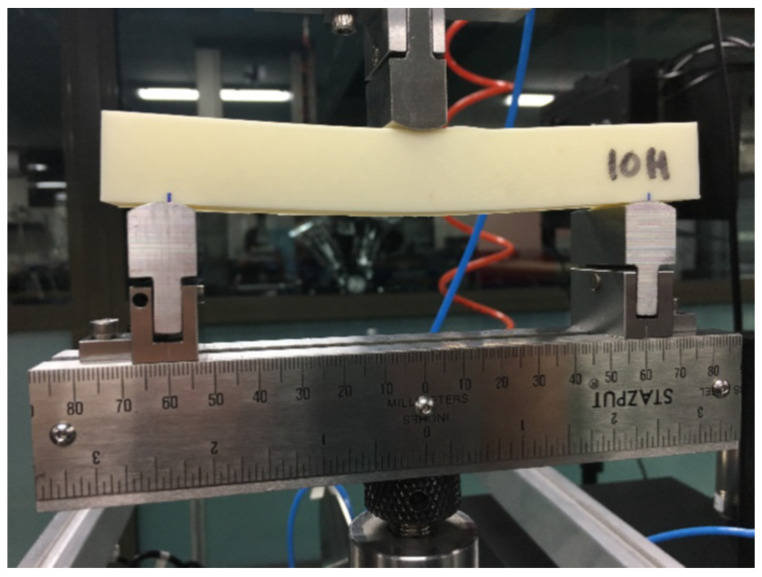
Bending test setting.

Six main configurations were manufactured varying their printing orientation and in-fill density, and several samples were produced for each case. Vertical (parallel to the Z axis) and horizontal (parallel to X axis) parts were printed, and for each orientation, we tested 10, 20, and 50% in-fill densities. The experimental setup for the horizontal 10% in-fill density (H-10) case is shown in Figure 5.

A rate of crosshead motion of 1.2 mm/min was set for the bending test performance, and the yield point was identified by means of the offset method for determining yield strength considering an offset value of 0.1%. Bending tests results are shown in Section 5 accompanied by their respective computational simulations.

## 4. Computational Characterization

### 4.1. Representative Volume Element

In order to save both printing time and material, the inner zone of an FFF part is conformed to a greater or lesser extent by air gaps, which leads to a complex geometry and a heterogeneous structure. Because of this, its high-fidelity discretization becomes cumbersome and expensive. In this context, computational homogenization becomes crucial. Homogenization methods that use the concept of the Representative Volume Element (RVE) have emerged as one of the most effective ways to address this issue.

**Figure 6:**
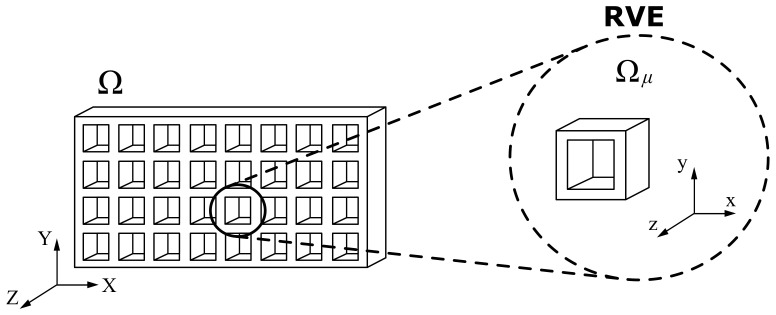
Representative volume element.

As explained in Section 1, two main different kinds of inner structures exist: in-fill and lattice. The present work was developed for the former one, and specifically for a cross-pattern at ±45°. The in-fill was heterogeneous but periodic, which implies the existence of a unit cell that repeats over the whole domain, the so-called RVE shown in Figure 6.

This methodology was previously used for inner zones in additively manufactured parts’ simulations with notable success [44]. The following section describes the computational homogenization process.

### 4.2. Multiscale Computational Homogenization

Homogenization of heterogeneous materials was performed within the framework of multiscale methods, where a scale distinction between the micro (RVE) and macro (the actual component) was made. The macro-scale was solved via a standard FE method, and then, these results were used as an input for solving the RVE at the micro-scale.

**Figure 7:**
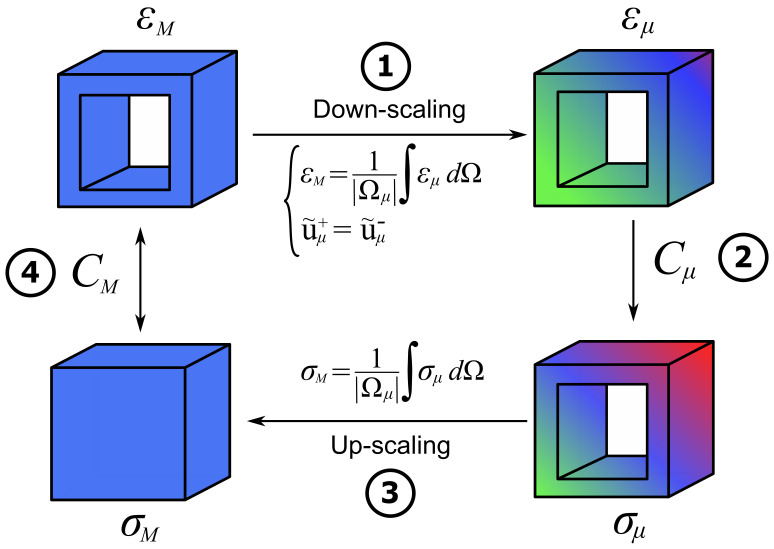
Homogenization procedure.

In this paper, First-Order Computational Homogenization (FOCH) was applied. Hereafter, subscripts *M* and μ denote the macro- and micro-scales, respectively. The objective was to compute the homogenized macro constitutive tensor $C_{M}$ of the in-fill material, and the idea derived from the decomposition of the micro displacement field $u_{\mu }$ as:

(4) $$u_{\mu }\left(x\right)=u_{M}\left(X\right)+{\tilde{u}}_{\mu }\left(x\right)$$

where $u_{M}$ stands for the macro displacements, ${\tilde{u}}_{\mu }$ for the micro displacements’ fluctuation, x denotes the coordinates at the micro-scale, and X at the macro-scale. Thus, the homogenization process was performed using a down-scaling process based on Equation (4), then solving the equilibrium equation of the RVE using the finite element method, and finally, up-scaling the results from micro to macro. Periodic Boundary Conditions (BCs) were applied at pairs of opposing faces of the RVE [45]:

(5) $${\tilde{u}}_{\mu }\left(x_{\mu }^{+}\right)={\tilde{u}}_{\mu }\left(x_{\mu }^{-}\right),\;\forall \;pairs\;\left\{x_{\mu }^{+},x_{\mu }^{-}\right\}$$

where $x_{\mu }^{+}$ and $x_{\mu }^{-}$ are pairs of points that belong to opposite faces of the RVE, which was considered to have a cubic geometry for this work. The whole procedure is depicted in Figure 7 and detailed in Algorithm 1, and additional details were given in [46,47,48,49,50,51,52,53]. The computational tool used for the in-fill homogenization was the in-house software Kratos Multiphysics [54].
**Algorithm 1:** First-order computational homogenization.
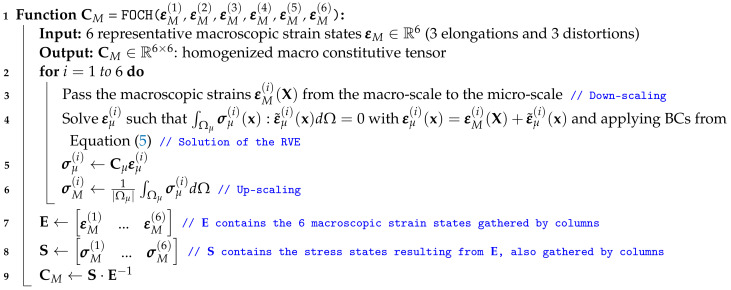


## 5. Experimental and Computational Results

### 5.1. Tensile Tests

**Figure 8:**
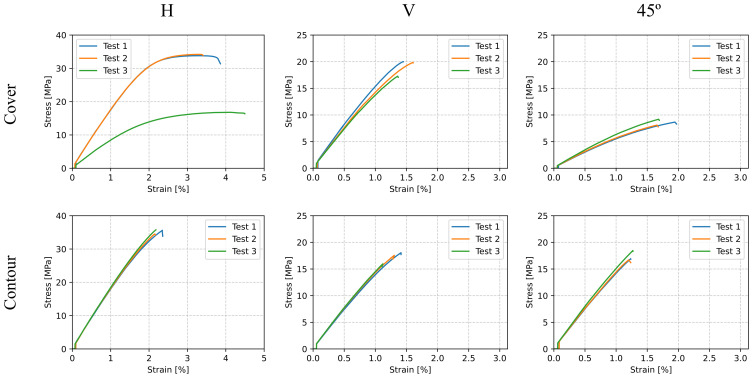
Tensile test stress–strain curves for ABS/PC.

The stress–strain curves resulting from the tensile tests described in Section 3.1 are plotted in Figure 8. Charts in the first row correspond to the cover zone samples, while the second row shows those of the contour zone. The distinction among the printing orientation was made in the different columns. For convenience, the axes for both the vertical and 45° cases were scaled according to their data range. Table 3 shows the corresponding material parameters for the cover and contour zones.

It can be seen from the stress–strain curves the Young’s moduli of the horizontally printed parts were the highest ones since their stiffness was measured along the direction of the extruded filament, and therefore, the mechanical properties of the part were close to those of the raw material. Parts printed in the horizontal direction showed the largest ultimate tensile strength and the largest corresponding strain. This was explained by the inter-layer unions, which reduced the part’s strength in the vertical direction because of the bigger influence of the bonding effect to the detriment of the properties of the material. It also confirmed the transversely isotropic behavior of 3D-printed parts and demonstrated the influence of the build orientation on their mechanical properties.

Only specimens from the cover zone and those horizontally oriented (H-Cover configuration) showed a ductile fracture, while the rest presented a brittle breaking. Non-horizontal samples showed brittle fracture because of the inter-layer adhesion phenomena, which led to failure caused by the separation of two contiguous layers. On the one hand, H-Cover samples presented yielding due to their non-aligned pattern in combination with the absence of remarkably influential inter-layer unions. On the other hand, H-Contour specimens were brittle because their filaments were aligned with the load application, and therefore, they behaved as standard polymers below 75% of their glass transition temperature $T_{g}$ [40].

### 5.2. Homogenization

Table 4 shows the results of the homogenized in-fill from the computational homogenizations explained in Section 4 for 10, 20, and 50% in-fill densities following a cross-pattern at ±45°. These results reflected the influence of the in-fill percentage, as well as the transverse isotropy shown by 3D-printed parts.

Young’s and shear moduli increased with the in-fill density, as expected. The fact that the apparent Poisson’s ratio in the XY plane (0.99 for the 10% in-fill density case) was almost three times higher than the one from the raw material (0.36) proved the existence of a strong geometrical influence in the mechanical properties of the final 3D parts. It should be noted that Poisson’s ratio between the transverse and in-plane directions ($\nu_{yz}$ and $\nu_{xz}$) was independent of the in-fill density, as shown in Table 4. Lastly, the XY plane (printing plane) clearly showed isotropic behavior.

### 5.3. Experimental Validation

**Figure 9:**
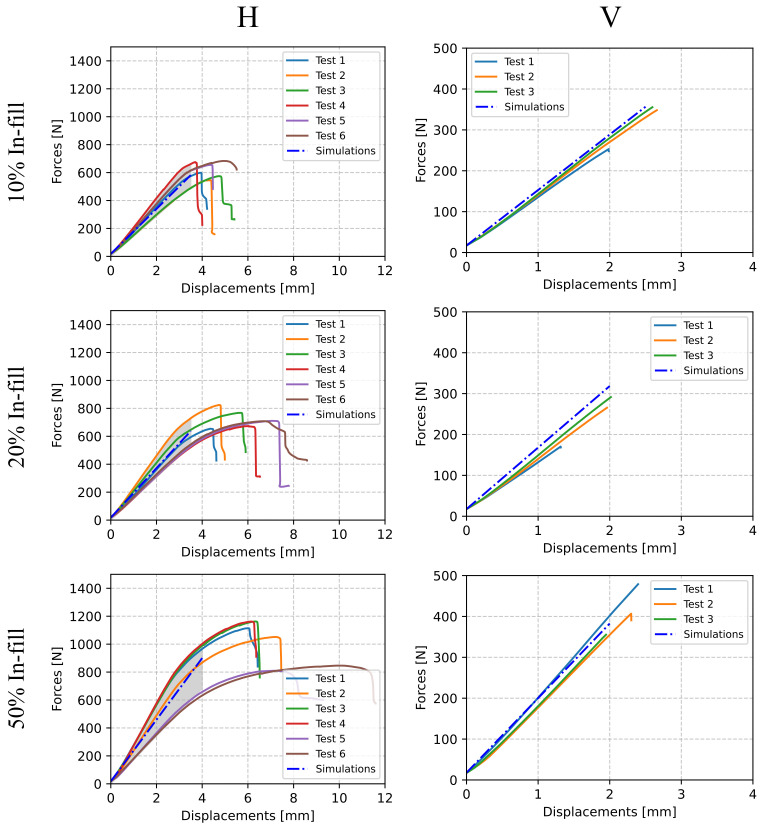
Bending test force–displacement curves for ABS/PC.

Figure 9 shows the force–displacement curves from the bending tests described in Section 3.2 along with their comparison with the simulation results within the elastic range. Right and left columns depict the horizontal and vertical cases, respectively, while each row represents a different in-fill percentage. For some configurations, the axes were scaled for convenience, similarly to the tensile test plots. Since a relatively wide dispersion was exhibited by these results, in particular the horizontally printed samples, a shaded area ranging from the minimum to the maximum obtained forces within the elastic range was plotted.

The experimental stiffness ($K_{experimental}$) was computed as the slope of the resulting force–displacement curve in the linear regime for each test, and the computational stiffness ($K_{simulation}$) was obtained also as the force–displacement ratio shown by the simulation data. These simulations were performed with Comet [55], an in-house software. Table 5 shows the obtained values for these stiffnesses, as well as their relative differences.

The effect of the printing orientation was also shown in the bending tests. It can be seen from Figure 9 that V samples suffered a brittle type of fracture, while H specimens presented ductile deformation. As for the tensile tests, this difference in the failure type was related to the inter-layer unions. The failure in the V samples was caused by an abrupt separation of two contiguous layers that resulted in a brittle fracture, while the H samples presented yielding because of the cross-pattern of the covers and the lower influence of the bonding effect. The same conclusion can be deduced from the stiffnesses, where the value of $K_{experimental}$ on the H samples was higher than on the V samples that were produced with the same in-fill density. As expected, an increase of the in-fill density increased both samples’ stiffness and strength.

Computational simulations were in remarkable agreement with experimental data. Figure 9 shows how the simulated force–displacement slope always fell within the range of the experimental results for H specimens in spite of their dispersion and was remarkably similar to the experimental data for the V cases. This agreement is also confirmed in Table 5, where the relative difference found between the computational and experimental stiffnesses was below 3% in the majority of cases.

## 6. Dimensional Analysis on the Three-Zone Model

**Figure 10:**
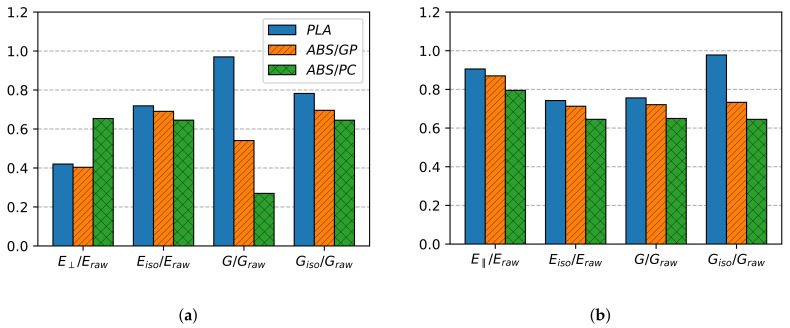
Non-dimensional relationships for PLA, ABS/GP, and ABS/PC referred to raw material properties. (**a**) Cover zone. (**b**) Contour zone.

For comparison, the mechanical properties of PLA, ABS/GP, and ABS/PC were expressed as a ratio to their respective raw material properties. Figure 10 shows the non-dimensional comparison for the cover and contour zones. The properties of PLA and ABS/GP were taken from the previous works [32,33], where the experimental methodology of this paper was used. Their non-dimensional properties were within the expected range, all ratios being below unity. For this case, they can also be called normalized properties since all of them were of the same order of magnitude [34]. Two main material properties groups were distinguished: the ones unaffected by inter-layer adhesion ($E_{iso}^{cover}$, $G_{iso}^{cover}$, $E_{\|}$) and the affected ones ($E_{⊥}$, $G^{cover}$, $E_{iso}^{contour}$, $G^{contour}$, $G_{iso}^{contour}$). This distinction was made a priori based on whether the inter-layer unions were stressed or not in their corresponding property test. For example, $E_{\|}$ was adhesion independent since the fibers, and consequently the inter-layer joining, were aligned with the applied force in the corresponding test. In contrast, layer unions in $E_{⊥}$ formed 90° with respect to the applied force in the test, and therefore, adhesion effects may emerge.

**Figure 11:**
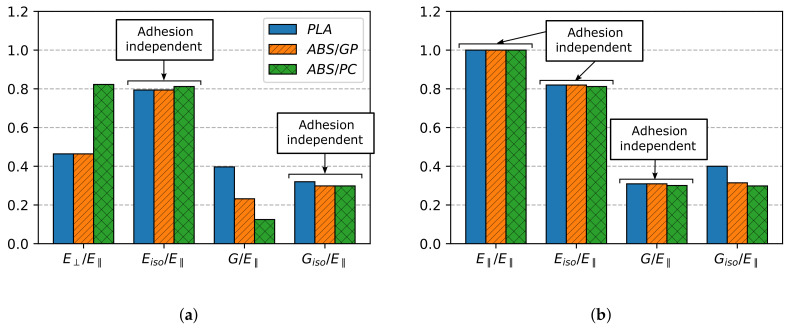
Non-dimensional relationships for PLA, ABS/GP, and ABS/PC referred to $E_{\|}$. (**a**) Cover zone. (**b**) Contour zone.

In relation to properties independent of inter-layer adhesion, it can be seen in Figure 10 that all of them showed similar values for the three tested materials, with the mechanical properties of PLA the highest ones and the ABS/PC properties the lowest ones. Figure 11 shows the same results, but referred to $E_{\|}$ instead, and there, it can be seen that these small variations vanished. With this procedure, the dispersion of the provider’s data was disregarded. Consequently, and according to Table 6, experiments with horizontally printed specimens are no longer required to characterize the aforementioned properties, and they can be computed using the non-dimensional relationships established in Table 7.

Moreover, regarding $E_{iso}^{contour}$ and $G^{contour}$, considered as potentially adhesion-affected properties, the adhesion effect on them was also negligible since their non-dimensional values did not vary for the three tested materials. Therefore, based on Table 6, it was confirmed that the 45-Contour tests could be omitted. In addition, V-Contour samples were not needed either since $G_{iso}^{contour}$ was obtained using Equation(3). Contrariwise, dimensionless properties $E_{⊥}$ and $G^{cover}$ varied for different materials, as shown in Figure 11a, which indicated that the physical process of adhesion was relevant.

In light of the above, the number of required experiments could be reduced to one third from the six initial configurations to just V-Cover and 45-Cover, a significant reduction that implies considerable cost savings.

**Figure 12:**
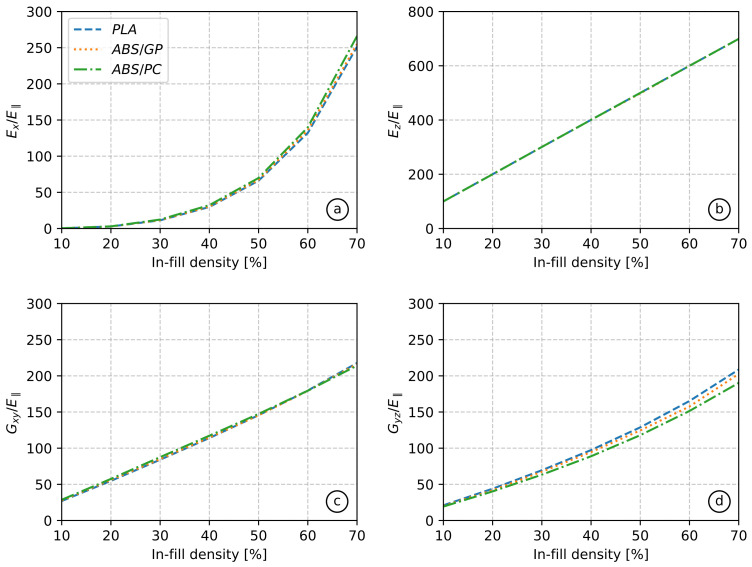
Non-dimensional relationships for the inner zone from all tested materials: Young’s modulus in direction X (**a**), Young’s modulus in direction Z (**b**), shear modulus in the XY plane (**c**), shear modulus in the YZ plane (**d**).

The non-dimensional values for the ±45° in-fill referred to $E_{\|}$ are depicted in Figure 12 as a function of in-fill density. Values corresponding to an in-fill density above 70% were not considered in order to avoid possible intra-layer phenomena. Poisson’s ratios were not plotted due to their lower influence on the material mechanical behavior. It can be seen that Young’s modulus in the Z direction showed a linear relationship with in-fill density, as expected, since stiffness in this direction and the area in the printing plane are directly proportional properties. In contrast, Young’s modulus in the isotropic plane showed a non-linear correlation with in-fill density due to the fact that the effective area of the RVE in X and Y directions had a more complex relation to in-fill density. Finally, it can be seen that the non-dimensional properties did not depend on the material. On account of that, computational homogenizations for the in-fill characterization can be replaced by charts as those in Figure 12, obtained experimentally or computationally.

Inter-layer adhesion needs to be further investigated in order to fully understand the mechanical behavior of FFF parts. In this context, the use of Scanning Electron Microscopy (SEM) imaging could help to evaluate the inter-layer bonding performance [56]. Up-to-date research suggests that major influential factors for inter-layer strength are a combination of both inter-layer contact and diffusion processes [57]. Coogan and Kazmer [58] developed a model that describes the interlayer bond strength of the parts in terms of two non-dimensional numbers: one related to the thermal diffusion across layers and the other quantifying the interlayer contact influence. This approach may be extended to characterize the influence of inter-layer strength in the elastic moduli of the AM components. Furthermore, Machine Learning (ML) is becoming increasingly used in the AM field for both properties’ prediction and optimization [59,60].

## 7. Conclusions

In this study, the mechanical properties of 3D-printed parts made of ABS/PC were characterized experimentally and computationally. The reported results put in evidence that the considered three-zone model was a reliable approximation for the characterization of 3D-printed parts and confirmed the direct impact of process parameters such as printing pattern and build orientation.

Results from tensile tests confirmed that both stiffness and strength at the inter-layer unions were lower than at the intra-layer joining and on the filament direction. The mechanical properties of horizontally printed parts were the closest to those of the raw material due to the absence of inter-layer bonding effects. Moreover, parts horizontally manufactured and with a cross-pattern showed the highest strength because they were the only specimens that underwent ductile fracture.

Results from bending tests reflected the influence of both the build orientation and the in-fill density on the mechanical performance of the 3D-printed part. An increase of in-fill density increased the stiffness of the component and decreased the influence of the build orientation on the mechanical properties of the part. The results from the computational simulations showed close agreement with the experiments and demonstrated the high fidelity of the three-zone model. Furthermore, the transversely isotropic behavior for cover, contour, and inner zones was verified.

It was concluded that non-dimensional relationships could be established for adhesion-independent mechanical properties, which implies that the number of testing configurations required to fully characterize the 3D-printed parts was reduced from six to two. These relationships must be referred to the 3D-printed part properties instead of the raw material ones to avoid manufacturing data dispersion.

Future research needs to address the better understanding of the inter-layer adhesion issue. If a quantitative relationship is established between adhesion-dependent properties and inter-layer stiffness properties, such as inter-layer contact or polymer chain diffusion, a significant decrease of the needed tests will be achieved through modelization or ML.

Finally, this research helped to establish new design-for-manufacturing techniques through the use of high-fidelity computational simulations. These low-cost simulations enable a high degree of customization of the parts by modifying printing parameters such as build orientation or in-fill density, optimizing their mechanical properties and subsequent performance.

## Figures and Tables

**Table 1:** Cover and contour properties’ nomenclature.

	$E_{x}$	$E_{y}$	$E_{z}$	$\nu_{\mathrm{xy}}$	$\nu_{\mathrm{yz}}$	$\nu_{\mathrm{zx}}$	$G_{\mathrm{xy}}$	$G_{\mathrm{yz}}$	$G_{\mathrm{zx}}$
Cover	$E_{iso}$	$E_{iso}$	$E_{⊥}$	$\nu_{iso}$	ν	ν	$G_{iso}$	*G*	*G*
Contour	$E_{\|}$	$E_{iso}$	$E_{iso}$	ν	$\nu_{iso}$	ν	*G*	$G_{iso}$	*G*

**Table 2:** Printing parameters.

Printing Parameter	Value
Extrusion temperature (°C)	250
Base temperature (°C)	95
Layer thickness (mm)	0.15
First layer printing speed (mm/s)	15
Contour printing speed (mm/s)	18
Cover printing speed (mm/s)	20
Inner printing speed (mm/s)	25

**Table 3:** ABS/PC properties for the cover and contour zones.

Material Properties	Cover	Contour
$E_{\|}$ (GPa) $E_{⊥}$ (GPa)	1.53 ± 0.09	1.86 ± 0.04
$E_{iso}$ (GPa)	1.51 ± 0.5	1.51 ± 0.03
$\nu_{iso}$	0.36 ± 0.01	0.36 ± 0.01
ν	0.36 ± 0.01	0.36 ± 0.01
*G* (GPa)	0.232 ± 0.02	0.559 ± 0.02
$G_{iso}$ (GPa)	0.555 ± 0.2	0.555 ± 0.01

**Table 4:** ABS/PC properties for the homogenized in-fill (cross-pattern).

In-Fill (%)	10	20	50
$E_{x}$ (MPa)	0.61	5.47	129.99
$E_{y}$ (MPa)	0.61	5.47	130.02
$E_{z}$ (MPa)	186.00	372.00	930.00
$\nu_{xy}$	0.99	0.9739	0.8022
$\nu_{yz}$	0.3528	0.3530	0.3530
$\nu_{xz}$	0.3532	0.3530	0.3530
$G_{xy}$ (MPa)	53.33	106.98	274.11
$G_{yz}$ (MPa)	35.84	74.83	219.43
$G_{xz}$ (MPa)	35.84	74.83	219.43

**Table 5:** Comparison between experimental and simulation results for the square cross-section bending test (ABS/PC material).

In-Fill Density (%)	Printing Orientation	$K_{\mathrm{experimental}}$ (N/mm)	$K_{\mathrm{simulation}}$ (N/mm)	Relative Difference (%)
10	H	174.33	161.18	7.54
	V	139.10	135.77	2.39
20	H	179.26	177.13	1.19
	V	139.61	150.49	7.79
50	H	223.25	220.67	1.16
	V	186.38	182.46	2.10

**Table 6:** Experiment set summary.

ASTM	Orientation	Pattern	Nomenclature	Characterized Property
ASTM D638	Horizontal	Crossed (cover)	H-Cover	$E_{iso}^{cover}$, $G_{iso}^{cover}$
		Aligned (contour)	H-Contour	$E_{\|}$
	Vertical	Crossed (cover)	V-Cover	$E_{⊥}$
		Aligned (contour)	V-Contour	$E_{iso}^{contour}$, $G_{iso}^{contour}$
	45°	Crossed (cover)	45-Cover	$G^{cover}$
		Aligned (contour)	45-Contour	$G^{contour}$
ASTM D790	Horizontal	In-fill 10%	H-10	
		In-fill 20%	H-20	
		In-fill 50%	H-50	
	Vertical	In-fill 10%	V-10	
		In-fill 20%	V-20	
		In-fill 50%	V-50	

**Table 7:** Non-dimensional relationships for adhesion-independent mechanical properties.

Property	Value
$E_{iso}^{cover}/E_{\|}$	0.80
$G_{iso}^{cover}/E_{\|}$	0.30
$E_{\|}/E_{raw}$	0.87
$E_{iso}^{contour}/E_{\|}$	0.82
$G^{contour}/E_{\|}$	0.31

## Data Availability

Data are contained within the article.

## Abbreviations

AM	Additive Manufacturing
MJF	Multi-Jet Fusion
SLM	Selective Laser Melting
EBM	Electron Beam Melting
FFF	Fused Filament Fabrication
FDM	Fused Deposition Modeling
PLA	Polylactic Acid
ABS/GP	General Purpose Acrylonitrile Butadiene Styrene
ABS/PC	Polycarbonate Acrylonitrile Butadiene Styrene
RVE	Representative Volume Element
FOCH	First-Order Computational Homogenization
BC	Boundary Condition
ML	Machine Learning
